# Motor Sequence Learning across Multiple Sessions Is Not Facilitated by Targeting Consolidation with Posttraining tDCS in Patients with Progressive Multiple Sclerosis

**DOI:** 10.1155/2021/6696341

**Published:** 2021-02-09

**Authors:** Harald Seelmann-Eggebert, Muriel Stoppe, Florian Then Bergh, Joseph Classen, Jost-Julian Rumpf

**Affiliations:** Department of Neurology, University of Leipzig, Leipzig, Germany

## Abstract

Compared to relapsing-remitting multiple sclerosis (MS), progressive MS is characterized by a lack of spontaneous recovery and a poor response to pharmaceutical immunomodulatory treatment. These patients may, therefore, particularly benefit from interventions that augment training-induced plasticity of the central nervous system. In this cross-sectional double-blind cross-over pilot study, effects of transcranial direct current stimulation (tDCS) on motor sequence learning were examined across four sessions on days 1, 3, 5, and 8 in 16 patients with progressive MS. Active or sham anodal tDCS of the primary motor cortex was applied immediately after each training session. Participants took part in two experiments separated by at least four weeks, which differed with respect to the type of posttraining tDCS (active or sham). While task performance across blocks of training and across sessions improved significantly in both the active and sham tDCS experiment, neither online nor offline motor learning was modulated by the type of tDCS. Accordingly, the primary endpoint (task performance on day 8) did not differ between stimulation conditions. In sum, patients with progressive MS are able to improve performance in an ecologically valid motor sequence learning task through training. However, even multisession posttraining tDCS fails to promote motor learning in progressive MS.

## 1. Introduction

Multiple sclerosis (MS) is a chronic, autoimmune inflammatory disease of the central nervous system (CNS) and the predominant cause of nontraumatic neurological disability in young adults [1, 2]. In the majority of cases, MS begins with alternating episodes of neurological impairment and subsequent recovery referred to as relapsing-remitting MS (RRMS) [3]. Following the initial relapsing and remitting disease course, approximately 60-70% of patients with MS (pwMS) who are initially classified as patients with RRMS later convert to a secondary progressive disease course (SPMS) that is characterized by a nonrelapse-related gradual progression of neurological decline without meaningful recovery [2–5]. In addition, about 10% of pwMS already demonstrate a nonrelapse-related gradual progression of functional impairment already at the onset of the disease, which is then referred to as primary progressive MS (PPMS) [3, 4]. Compared to RRMS, both primary and secondary progressive forms of the disease are characterized by a lack of spontaneous recovery and a poor response to pharmaceutical immunomodulatory treatment [6]. Apart from the extent of the accumulating MS-induced myelin loss and neuronal injury, MS-associated functional impairment probably depends on the ability of the central nervous system to compensate the ongoing neuronal injury [7]. One mechanism that may be able to mitigate MS-associated functional disability is the induction of CNS plasticity by repeated physical training. Given the poor response to current immunomodulatory pharmaceutical interventions and the absence of spontaneous recovery in PPMS and SPMS, these patients may particularly benefit from interventions that are able to augment training-induced CNS plasticity to support functional compensation for the ongoing accumulation of neuronal damage.

In the motor domain, the acquisition of new skills as well as the reacquisition of lost motor skills due to brain injury is driven by repeated skill training and evolves across online and offline learning stages that are sustained by distinct mechanisms [8, 9]. The initial “online” learning phase during which the skill to be learned is repeatedly executed for the first time is followed by an “offline” phase that leads to a transformation of the initially labile motor memory into a more robust representation in the absence of further training [10, 11]. Performance of the newly acquired motor skill may then be further enhanced by additional cycles of online and offline learning that ultimately enable smooth and effortless execution of the trained skill [9]. Remarkably, although overall motor task performance is compromised in pwMS compared to healthy subjects, the “online” process of motor learning (i.e., the relative skill improvement across a training session as a function of practice) seems to be relatively spared from disease-associated impairment [12–14]. Against this background, the functional impact of motor training in MS may eventually be determined by the ability to stabilize (i.e., consolidate) skill improvements that were acquired online. Facilitating posttraining offline consolidation processes may, therefore, represent a reasonable target to aid functional compensation of motor skill impairments due to MS-associated neural damage.

Noninvasive brain stimulation by transcranial direct current stimulation (tDCS) has been demonstrated to modulate neuronal excitability and behaviour in healthy human subjects as well as in patients with brain injury [15]. With respect to effects on motor learning, a large body of evidence supports that tDCS effectively promotes training-induced skill improvements across a training intervention (i.e., online learning), when task training is combined with concomitant tDCS of the primary motor cortex (M1) (e.g., [16–19]). Moreover, tDCS was also shown to beneficially interact with the offline consolidation process after training. Facilitation of consolidation may be either induced by the application of tDCS “online” during training [20, 21] or “offline” after termination of the training intervention [22–24]. In MS, however, neither single sessions of online tDCS during motor training [25] nor single sessions of offline application of tDCS following motor skill practice [12] facilitated online learning or offline consolidation. Given the evidence pointing to impaired susceptibility of the motor system of pwMS to a single session of tDCS, we here investigated whether multiple sessions of motor training combined with posttraining offline tDCS will specifically improve consolidation and, thus, overall motor learning across sessions in patients with progressive MS.

## 2. Methods

### 2.1. Ethical Standards

The study protocol was approved by the institutional ethical standards committee at the University of Leipzig (registration code, 033/17-ek) and was registered at the German Clinical Trials Register (DRKS, registered on 03 May 2018; ID: DRKS00014598). All methods were performed in accordance with the relevant guidelines and regulations and all participants provided written informed consent before study-related procedures were conducted.

### 2.2. Participants

Participants were recruited via the neuroimmunology outpatient clinic of the Department of Neurology at the University of Leipzig. Out of 145 patients with primary or secondary progressive MS that were prescreened for potential eligibility, finally 16 patients (PPMS, *n* = 10; SPMS, *n* = 6) aged between 33 and 64 years (51.2 ± 10.6 years, mean ± SD, 6 females) were recruited and included in the study. Two additionally screened subjects were not included as they did not match inclusion/exclusion criteria. Disease duration (defined as years since diagnosis of progressive MS) ranged from 1 to 32 years (13.1 ± 9.6 years). Inclusion criteria comprised age between 18 and 65 years, definite diagnosis of PPMS or SPMS according to the 2010 McDonald Criteria [26], a current Expanded Disability Status Scale (EDSS, [27]) score between 0 and 6.5, and right handedness according to the Edinburgh Handedness Inventory [28]. SPMS participants had to be without relapse for more than one year. Exclusion criteria included pregnancy, treatment with 4-aminopyridine within the last three months, onset of a disease modifying therapy within the last three months, other CNS diseases than progressive MS, current use of sedatives, and contraindications for tDCS (e.g., history of epileptic seizures, implanted electrical devices). None of the participants were (semi-)professional musicians or had been trained as a typist. All participants underwent a full neurological examination including the assessment of the EDSS. Fatigue was assessed using the Würzburger Fatigue Inventory for MS (WEIMuS, [29]), and symptoms of depression were assessed by the Beck Depression Inventory (BDI, [30]). The 9-hole peg test (right hand), the timed 25-foot walk test, and the spoken Symbol Digit Modalities Test (SDMT, [31]) were completed on the initial and the last session of each of both experiments to assess potential changes in fine motor function of the trained hand, walking speed, and cognitive processing.

### 2.3. Experimental Design

**Figure 1:**
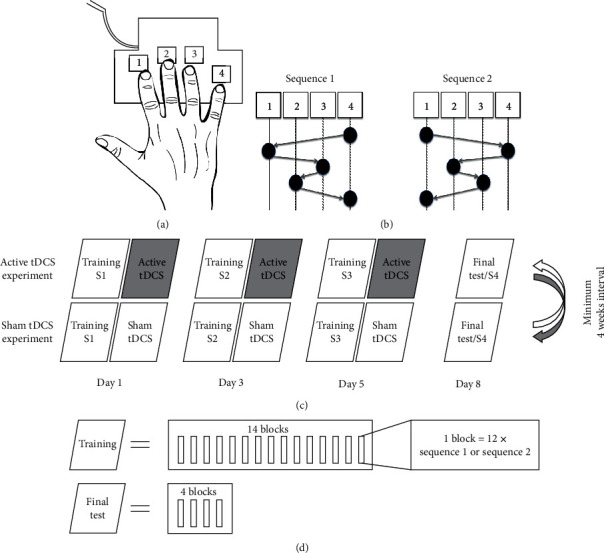
Experimental design. (a) The explicit motor sequence learning task was executed on a four-button gaming keyboard with the right hand. (b) Participants practiced either one of two equally difficult sequences in the active tDCS experiment and the sham tDCS experiment (balanced in terms of order and assignment to the active or sham condition). (c) All participants took part in two experiments, which were separated by at least 4 weeks and corresponded to two types of posttraining tDCS interventions, i.e., active anodal tDCS of the left primary motor cortex and sham tDCS. Each experiment encompassed three training sessions on day 1 (S1), day 3 (S2), and day 5 (S3) and a final test on day 8 (S4). Posttraining tDCS was applied with the anode placed over the left primary motor cortex and the cathode placed over the right supraorbital region. (d) Each training session consisted of 14 blocks of task training, in which each encompassed the execution of 12 sequences (i.e., 60 button presses). The final test consisted of four blocks of the task.

All participants took part in two experimental sets in a cross-over design that corresponded to two different types of posttraining tDCS intervention, i.e., posttraining sham tDCS and posttraining active tDCS. Experiments were separated by at least four weeks to minimize carry-over effects and were balanced across participants with respect to the order of the type of the posttraining tDCS intervention in the first and second experimental set. Both experimental sets were, furthermore, balanced with respect to the two different but equally difficult finger movement sequences applied in experimental sets 1 and 2. Participants were sequentially recruited and allocated to a combination of sequence of experiments (Expt. 1=active/Expt. 2=sham or Expt. 1=sham/Expt. 2=active tDCS) and sequence of applied finger movement sequences (Expt. 1=sequence 1/Expt. 2=sequence 2 or Expt. 1=sequence 2/Expt. 2=sequence 1) in a pseudorandomized manner using predefined lists to assure balancing of factors. Each of both experimental sets consisted of three training sessions on day 1, day 3, and day 5, and a final assessment of motor task performance on day 8. All training sessions as well as the final assessment of motor task performance were performed in the morning between before 12 a.m. to limit the influence of potential circadian fluctuations of motor performance (Figure 1).

### 2.4. Motor Sequence Learning Task

Motor sequence performance was assessed by an adapted version of the sequential finger-tapping task introduced by Karni and colleagues [10]. For each training session as well as for the final assessments of task performance, participants were instructed to execute a five-element finger-tapping sequence on a customized four-button gaming keyboard with their right (dominant) hand. Two different but equally difficult sequences were used in experimental sets 1 and 2 (balanced across participants/order of experiments): sequence 1: “4-1-3-2-4”; sequence 2: “1-4-2-3-1” (where 1 = index finger, 2 = middle finger, 3 = ring finger, and 4 = little finger). Participants had to demonstrate explicit knowledge of the respective sequence prior to each training session by slowly repeating the sequence three times in a row without making a mistake. Each training session consisted of 14 consecutive blocks of sequence execution that were separated by rest periods that lasted 25 seconds (Figure 1). Prior to each training session, participants were instructed to execute the sequence as fast as possible while making as few errors as possible. Each task block was automatically terminated after 60 key taps to ensure that all participants received the same amount of training (i.e., executed the same number of finger movements). This implies that a maximum of 12 correctly executed sequences could be contained within each training block. Onset of a training block was indicated by a green fixation cross in the middle of a computer screen on a desk in front of the participants, which changed its color to red to indicate the onset of a rest block. No information on the sequence was presented to the participants during training or rest blocks. The final assessment of task performance on day 8 in both experiments consisted of only 4 blocks of the task. Participants were instructed not to practice the sequence in-between sessions to prevent confounding offline consolidation with additional task training.

### 2.5. Posttraining Transcranial Direct Current Stimulation

Posttraining active or sham tDCS was applied for 15 minutes immediately following termination of each training session on days 1, 3, and 5 of both experimental sets. Setup of the direct current stimulator (DC-Stimulator-Plus, Neuroconn, Germany) and montage of the stimulation electrodes were completed before onset of the following training session in order to start the stimulation immediately after termination of the training session. The anode (5 x 5 cm) was centered at C3 (according to the 10-20 EEG system), which corresponds to the hand area of the left primary motor cortex. The cathodal electrode (5 x 5 cm) was placed on the right supraorbital region (i.e., ipsilateral to the trained hand). Electrodes were covered by sponges soaked in 0.9% sodium chloride solution. At stimulation onset, direct current was increased ramp-like for 8 seconds until the final stimulation current of 1 mA was reached (i.e., current density 0.04 mA/cm^2^). The sham stimulation procedure started identically, but the stimulation current faded out 30 seconds after reaching 1 mA. This method was shown to ensure successful blinding of the participants [32, 33]. The experimenter was also blind for the type of the current tDCS intervention as we used the implemented “study mode” of the stimulator that allows to use predefined codes to encode sham and active stimulation mode. For the duration of the stimulation, participants were instructed to relax and to watch a set of landscape photographs (photographs changed every 30 seconds) displayed on the computer screen in front of them.

### 2.6. Data Acquisition and Analysis

Timing of finger taps during sequence execution was recorded with a customized four-button gaming keyboard and processed using customized MATLAB (MathWorks, Natick, USA) scripts in order to extract the speed and the accuracy of sequence execution. Speed performance of sequence execution was defined as the average time that was required to perform a correct sequence within a given block of the task (average time to perform a correct sequence, TCS). Accuracy was defined as the number of correct sequences per block (i.e., a maximum of twelve correct sequences per block). Repeated measures analysis of variance (rmANOVA) with the within-subject factors *block* (B1,…, and B14), training *session* (e.g., S1, S2, S3, and S4), and tDCS *intervention* (i.e., sham vs. active) was applied to speed and accuracy measures to assess effects of repeated training and the stimulation intervention on task performance. The primary outcome measure was task performance in session 4. Secondary outcome measures were changes in the performance in the 9-hole peg test and task performance changes during online and offline stages of motor learning. Online learning across a training session was operationally defined as the difference of speed/accuracy performance between the beginning of a training session (BOT: average speed/accuracy performance of the first two blocks of the training session) and the performance at the end of that training session (EOT: average speed/accuracy of the last two blocks of the training session). Averaging across two blocks to calculate BOT and EOT was done to prevent that the online and offline learning measures were inflated or deflated due to performance fluctuations in single blocks at the beginning (e.g., warming up) or end (e.g., fatigue) of a training session. To quantify offline consolidation between sessions, we computed the difference between each individual's performance at EOT of a training session and the BOT performance of the following session. Note that online and offline performance changes were computed such that positive values indicated improved speed and accuracy performance across training and between sessions. Total online and total offline learning was then computed as the sum score of the three individual online learning measures across S1, S2, and S3 and the three offline consolidation measures assessed between S1/S2, S2/S3, and S3/S4. A single missing TCS value for one training block of participant 14 due to zero correctly performed sequences in that block (session 2, block 9 of the posttraining active tDCS experiment) was replaced by the mean TCS of the previous and the following block. In case of violation of the sphericity assumption, the Greenhouse-Geisser corrections were applied. Total online and offline learning values were compared between types of the posttraining tDCS intervention using paired-sample *t*-tests. Speed and accuracy measures are reported as mean with 95% confidence interval (CI). Spearman's rank correlation coefficient was applied to assess associations of motor performance, learning, and consolidation with clinical and functional characteristics. The alpha level for the correlation analyses was set to *p* < 0.01. For all other statistical tests, the alpha level was set to *p* < 0.05. All statistical analyses were carried out with SPSS 25 (SPSS, Chicago, IL, USA).

## 3. Results

Demographic information and clinical characteristics of the 16 individual participants are detailed in Table 1. All of the following analyses included data from all (*n* = 16) of these participants. There were no significant differences for any of the assessed clinical and functional characteristics between the posttraining active tDCS and sham tDCS experimental sets (paired *t*-tests, all *p* > 0.26). Of the functional tests that were assessed at baseline and at the last session (S4), only averaged performance (mean of performance across the active tDCS and the sham tDCS experiment) in the 9-hole peg test differed significantly between baseline and S4. This was driven by faster execution at S4 (mean ± SD, 27.4 ± 11.7 s) compared to baseline (28.7 ± 12.5 s, *p* = 0.020). Stimulation side effects consisted in sensations described as a tingling or an itching under the stimulation electrodes. Eight of the 16 participants were able to correctly identify the order of the sham and active tDCS intervention experiments after completion of both experimental sets suggesting successful blinding.

### 3.1. No Modulation of Overall Motor Learning by Posttraining tDCS

Overall motor learning was assessed by comparing mean task performance in terms of speed and accuracy in session 4 (day 8), which was preceded by either 3 training sessions (on day 1, 3, and 5) combined with posttraining sham or active tDCS of M1. rmANOVA applied to mean speed performance in session 4 (mean TCS across the 4 blocks of the task) showed no significant main effect of the within-subject factor *Intervention* (*F*_(1, 15)_ = 0.974, *p* = 0.339) indicating that overall motor learning was not modulated by posttraining tDCS. Average TCS across blocks in session 4 amounted to 1982 ms (CI 1561–2402) when prior training sessions were followed by posttraining sham tDCS and to 2126 ms (CI 1498–2754) when task training was combined with posttraining active tDCS of M1.

Similar results were obtained with respect to accuracy of task performance during session 4. Mean number of correct sequences per block (maximum 12) in session 4 amounted to 11.59 (sham tDCS, CI 11.39–11.80) and 11.41 (active tDCS, CI 10.86–11.96) and did not differ significantly between stimulation conditions (*Intervention*: *F*_(1,15_ = 0.622, *p* = 0.443). This, collectively, indicates no beneficial effect of repeated sessions of task training combined with posttraining anodal tDCS of M1 on motor learning across sessions in patients with progressive MS.

### 3.2. Online Learning during Training Sessions—Speed

**Figure 2:**
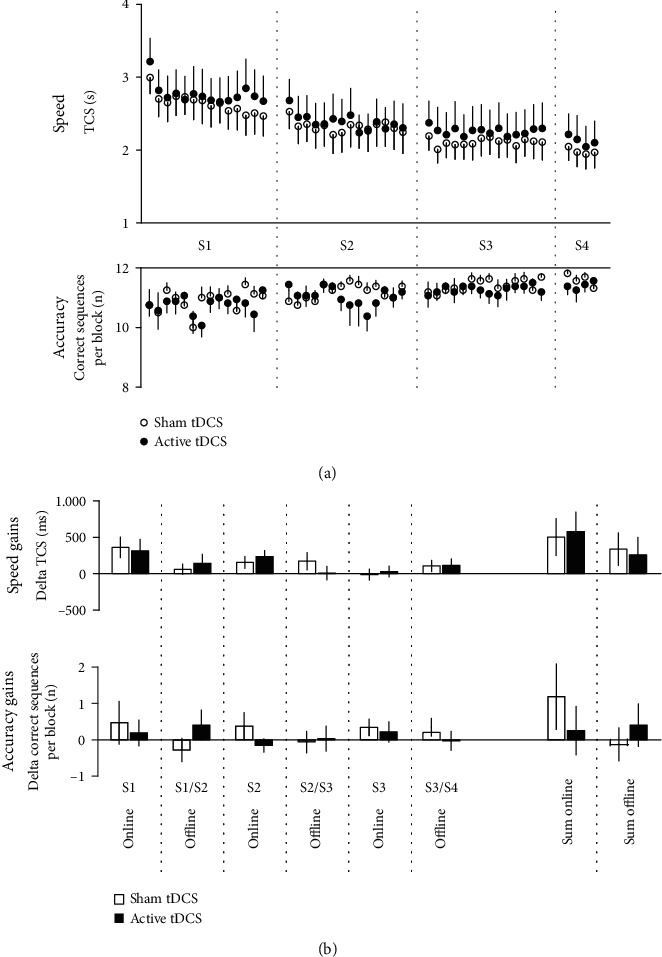
Speed and accuracy of task performance. (a) Speed (mean time to perform a correct sequence per block, TCS) and accuracy (number of correct sequences per block) of task performance across blocks of training in session 1 (S1) on day 1, session 2 (S2) on day 3, session 3 (S3) on day 5, and session 4 (S4) on day 8 of the posttraining sham and active tDCS experiments. Vertical bars represent the standard error of the mean (SEM). (b) Online and offline task performance changes. Online speed and accuracy performance changes during training sessions (S1, S2, and S3) were calculated as the difference of performance between the beginning of a training session (first two blocks) and the end of a training session (last two blocks) such that positive values indicate improvement of speed or accuracy performance across the training session. Offline performance changes between training sessions S1 and S2, S2 and S3, and S3 and S4 were assessed between the last two blocks of the previous training session and the first two blocks of the next training session such that positive values indicate offline speed and accuracy improvement of performance relative to the performance at the end of the previous session. Overall online and offline learning (sum online, sum offline) was calculated as the sum score of the three individual online (during session) and offline (between sessions) measures. Bars represent SEM.

rmANOVA conducted on the speed measure (TCS) with the within-subject factors *Block* (B1,…, and B14), *Session* (training sessions 1, 2, and 3), and posttraining *Intervention* (sham tDCS vs. active tDCS) revealed a significant main effect of *Block* (*F*_(2.5,37.6)_ = 5.039, *p* = 0.007) and *Session* (*F*_(1.3,19.4)_ = 32.054, *p* < 0.001) in the absence of a significant main effect of *Intervention* (*F*_(1, 15)_ = 0.670, *p* = 0.426) or a significant interaction of factors (all *p* ≥ 0.563). TCS at BOT of the first training session was similar (*p* = 0.256) in the posttraining sham tDCS experiment (2848 ms, CI 2365–3331) and the active tDCS experiment (3017 ms, CI 2386–3648) and reached 2114 ms (CI 1608–2621, sham tDCS) and 2292 ms (CI 1565–3020, active tDCS; *p* = 0.251) at EOT of the third training session (Figure 2(a)). In sum, this indicates similar online learning across blocks of practice and across training sessions irrespective of the type of the posttraining tDCS interventions. Accordingly, the overall online learning measure (sum of ΔBOT–EOT in sessions 1, 2, and 3) did not significantly differ between the posttraining sham tDCS intervention experiment (total online-generated improvement: 503 ms, CI -40–1045) and the active tDCS intervention experiment (577 ms, CI 5 ms–1149, *p* = 0.785; Figure 2(b)). Notably, rmANOVA further revealed a significant *Block* x *Session* interaction (*F*_(26,390)_ = 1.766, *p* = 0.013), which was driven by decreasing online improvements across repeated training sessions indicating asymptotic learning in training session 3 (mean (sham and active tDCS experiment) online improvement (ΔBOT–EOT): training 1: 337 ms, CI 84–591; training 2: 195 ms, CI -34–356; training 3: 7 ms, CI -121–136). This interpretation is further supported by the fact that, when rmANOVA was applied to the three training sessions separately, it revealed a significant main effect of *Block* in training sessions 1 (*F*_(3.2,47.9)=_3.651, *p* = 0.017) and 2 (*F*_(3.1,46.7)=_3.484, *p* = 0.022) while the main effect of *Block* was not significant in training session 3 (*F*_(3.2,48.1)=_1.212, *p* = 0.316).

### 3.3. Online Learning during Training Sessions—Accuracy

A similar rmANOVA conducted on the accuracy measure (number of correct sequences per block) showed a significant main effect of *Session* (*F*_(1.3,19.7)_ = 10.385, *p* = 0.002) in the absence of a significant main effect of *Block* (*F*_(3.6,53.9)_ = 1.115, *p* = 0.356) and posttraining *Intervention* (*F*_(1, 15)_ = 1.000, *p* = 0.333). There were no significant two-way interactions for any of the factors nor a significant three-way interaction of *Intervention* x *Block* x *Session* (all interactions *p* ≥ 0.433). Although mean accuracy across the first session was already high (sham and active tDCS experiments combined: 10.83, CI 10.29–11.38) and, thus, close to ceiling performance (maximum number of correct sequences per block = 12), it still improved gradually in the second (11.11, CI 10.66–11.57) and third training session (11.34, CI 11.00–11.67), which drove the significant main effect of the factor *Session* (Figures 2(a) and 2(b)). Collectively, while speed performance increased during online learning and across training sessions, accuracy of task performance only gradually improved between sessions but remained stable at a high level across blocks within training sessions (i.e., no relevant speed accuracy trade-off).

Similar to speed performance, accuracy during online learning did not differ between the series of posttraining sham and active tDCS interventions. Taken together with the speed performance results, this rules out that potential effects of the posttraining tDCS intervention on offline consolidation were confounded by differences of motor engram formation during online learning.

### 3.4. Consolidation between Sessions—Speed

Overall speed improvements (i.e., reduction of TCS) that were generated offline between sessions (sum of differences EOT–BOT between sessions) amounted to 337 ms (CI -134–809) when training sessions were followed by sham tDCS and to 260 ms (CI -248–768) when training sessions were followed by active tDCS and did not significantly differ between stimulation conditions (*p* = 0.768). Accordingly, rmANOVA across the three single consolidation measures (between sessions S1/T2, S2/T3, and S3/T4) revealed no significant main effect of *Intervention* (*F*_(1, 15)_ = 0.091, *p* = 0.768). Furthermore, rmANOVA showed no significant main effect of *Session* (*F*_(2, 30)_ = 0.047, *p* = 0.954) and no significant interaction of posttraining *Intervention* x *Session* (*F*_(2, 30)_ = 1.122, p =0.339; Figure 2(b)). This, collectively, suggests that the magnitude of offline performance changes between sessions in terms of speed were comparable across the experiment and were not modulated by posttraining tDCS.

### 3.5. Consolidation between Sessions—Accuracy

The overall offline change between sessions (sum of between-session offline changes) with respect to the number of correct sequences per block was comparable between posttraining sham and active stimulation (*p* = 0.566) and amounted to -0.13 correct blocks (CI -1.13–0.88) when training sessions were followed by sham tDCS and to 0.41 correct blocks (CI -0.84–1.65) when training sessions were followed by active tDCS (Figure 2(b)). rmANOVA across the three single consolidation measures between sessions showed no significant main effect for *Intervention* and *Session* as well as no significant interaction of factors (all *p* values ≥ 0.275). This indicates that, similar to the results for speed performance, task performance in terms of accuracy was not modulated offline between sessions by the posttraining tDCS intervention.

### 3.6. Correlations

Nonparametric (Spearman's rho) correlation analyses were calculated to assess associations of clinical characteristics (age, EDSS, years since diagnosis of progressive MS, BDI, and CGI-S) and functional tests (9-hole peg test, timed 25-foot walk, SDMT at baseline) with task performance measures (mean speed and accuracy performance across all blocks and sessions, sum scores of online and offline learning for speed and accuracy). As the above analyses revealed no significant differences between any of the motor sequence task performance and clinical/functional measures between the posttraining active tDCS experiment and the sham tDCS experiment, mean values of measures that were assessed twice (e.g., online learning sum score, offline learning sum score, BDI, CGI-S, 9-hole peg test, timed 25-foot walk, and SDMT) were entered into the analysis.

Not surprisingly, lower average TCS across all sessions (i.e., faster task execution) was significantly correlated with faster performance in the 9-hole peg test (rho = 0.747, *p* = 0.001). Moreover, lower average TCS and lower consolidation in terms of speed performance were associated with higher scores in the SDMT (rho = −0.722, *p* = 0.002; rho = −0.699, *p* = 0.003), suggesting a relevant role of cognitive function for motor sequence execution and consolidation. Lower accuracy of overall task performance was significantly correlated with higher age (rho = −0.657, *p* = 0.006) and severity of the patient's illness as rated by the CGI-S (rho = 0.645, *p* = 0.007). There were no significant associations of the total online and offline learning sum scores in terms of speed and accuracy performance with other clinical characteristics (age, EDSS, years since diagnosis of progressive MS, BDI, and CGI-S scores).

## 4. Discussion

The present study showed that patients with substantial disability due to progressive MS are well capable of improving motor sequence performance across multiple training sessions but that this is not modulated by immediate posttraining anodal tDCS directed to the primary motor cortex (M1). Training-induced performance increments within training sessions were similar for the active and sham posttraining tDCS intervention sessions. This excludes that potential effects of posttraining tDCS on offline consolidation could have been confounded by differences in motor engram formation during online learning. However, we also found no relevant effect of posttraining tDCS on offline speed or accuracy performance during the consolidation phases between sessions, which were specifically targeted by the stimulation protocol. Since all participants significantly improved task performance across repeated sessions of training, failure to promote consolidation by the posttraining tDCS intervention cannot be explained by an inherent inability of the study population to improve task performance through training. Instead, it suggests that the process of offline motor consolidation was not susceptible to modulation by tDCS.

These current findings in patients with progressive MS are in line with results of a previous single-session offline tDCS study that demonstrated compromised tDCS-induced facilitation of consolidation following explicit motor sequence training in patients with relapsing-remitting MS [12]. However, those previous findings as well as the current results contrast with several studies in healthy young and older subjects that reported modulation of consolidation by offline application of tDCS after motor sequence training [12, 22–24, 34]. Moreover, in addition to the failure to promote offline consolidation in pwMS, single-session anodal tDCS directed to the primary motor cortex also failed to facilitate online motor sequence learning in pwMS when applied concurrently with task execution [25], a protocol that frequently facilitated motor sequence learning in healthy subjects in previous studies [16, 18, 19, 35]. In sum, this body of evidence may point to disease-inherent properties of MS that render these patients' motor system insusceptible to the effects of tDCS of M1 with respect to online and offline motor sequence learning processes.

Results of previous studies suggested that repeated application of anodal tDCS to M1 concurrently with motor sequence training across five consecutive sessions may enhance the effects of a single-session tDCS intervention on online motor sequence learning [36] and promote overall learning by an effect on offline consolidation [21]. We are not aware of studies that investigated the application of tDCS concurrently (i.e., online) with multiple sessions of motor training in pwMS. Thus, there is no information on whether the absent tDCS effect on online motor sequence learning in MS observed during a single training session by Meesen and colleagues [25] may yet evolve across a multisession approach. However, current findings clearly indicate that in terms of specific facilitation of motor consolidation in pwMS, even multiple sessions of motor sequence training combined with posttraining offline tDCS are not sufficient to induce relevant effects on consolidation of training-induced speed or accuracy performance increments.

What may be the underlying pathophysiological mechanisms that render patients with relapsing-remitting [12] and progressive MS (in the current study) insusceptible to the effects of tDCS on motor sequence learning? Previous studies in healthy young subjects demonstrated that the induction of offline motor consolidation following explicit motor sequence learning was related to posttraining corticospinal excitability [37, 38]. Moreover, it was reported that offline consolidation following motor sequence training could be facilitated by remotely applying theta burst stimulation in order to increase corticospinal excitability immediately after a motor sequence training session [38]. Facilitation of consolidation by posttraining application of anodal tDCS of M1 in healthy young [12, 22, 24] and healthy elderly subjects [23] may have been induced by a similar mechanism (i.e., by preventing a posttraining decrease of corticospinal excitability in M1). This may imply that insusceptibility to the effects of posttraining tDCS on consolidation in pwMS could be related to a disease-associated impairment of modulation of corticospinal excitability by tDCS. However, previous studies showed that neither facilitation of corticospinal excitability by anodal tDCS of M1 [39] nor recruitment of LTP-like and LTD-like plasticity in M1 by transcranial magnetic stimulation differed between pwMS and healthy controls [7, 14]. This body of evidence suggests that the prerequisites to induce regional plasticity or enhance local use-dependent plasticity in M1 by noninvasive brain stimulation techniques are fundamentally intact in pwMS. However, besides local plasticity, online and offline stages of motor sequence learning rely on the dynamic recruitment of nodes of a distributed motor learning network that includes M1, the premotor cortex, the supplementary motor area, the cerebellum, basal ganglia, the hippocampus, and parietal cortical areas [40–45]. A recent fMRI study showed that alterations of sequence-specific motor learning in young healthy subjects induced by cerebellar tDCS were related to altered activity in M1, the cerebellum, the inferior frontal gyrus, and the right parietal lobule [46] suggesting that tDCS facilitated motor sequence learning by alterations of long-range network communication. Long-range network connectivity may be compromised in pwMS by both disease-related white matter lesions and affection of grey matter [47]. Thus, impaired susceptibility of pwMS to effects of tDCS of M1 in terms of modulation of online and offline motor sequence learning might be associated with a disease-related alteration of tDCS-induced motor learning network recruitment that underlies the beneficial effects of tDCS on motor sequence learning in healthy subjects. However, as a limitation of our study (besides the relatively low number of participants), disease-specific deficits of pwMS in terms of susceptibility to tDCS effects on motor learning can only be inferred from the above body of evidence in healthy subjects as no healthy control group was collected in our study.

We found that fine motor hand function as assessed by the 9-hole peg test improved significantly in pwMS from baseline to the assessment at day eight, suggesting some degree of generalization of the performance improvement in the motor sequence task acquired across repeated training sessions. As this effect was specific for the 9-hole peg test and not seen for the 25-foot walk test or the SDMT (assessing working memory and executive function), one might speculate that training-induced improvements of motor sequence performance specifically generalized to another fine motor hand function. However, in terms of our primary goal to facilitate the success of repeated motor sequence training by promoting the offline consolidation phase with noninvasive brain stimulation, posttraining anodal offline tDCS of M1 failed to modulate offline processing of training-induced performance increments.

## 5. Conclusions

Our findings demonstrate that posttraining anodal offline tDCS of M1 failed to improve offline consolidation even when applied over multiple sessions. This result implicates that pwMS lack regional susceptibility to the effects of tDCS on motor sequence learning. Future studies may target multiple brain regions to investigate whether more network-oriented stimulation protocols can address the dysfunctional long-range network connectivity in pwMS and facilitate motor learning through noninvasive brain stimulation.

## Figures and Tables

**Table 1:** Demographic and clinical characteristics of participants.

Participant	Age, y	EDSS, score	MS type	Disease duration, y	CGI-S, score		WEIMuS, score		BDI, score		T25W at baseline, s		9HPT at baseline, s		SDMT at baseline, score		T25W at session 4, s		9HPT at session 4, s		SDMT at session 4, score	
					atDCS	stDCS	atDCS	stDCS	atDCS	stDCS	atDCS	stDCS	atDCS	stDCS	atDCS	stDCS	atDCS	stDCS	atDCS	stDCS	atDCS	stDCS
1	51	4.0	PPMS	3	4	4	8	13	3	11	6.77	6.16	28.95	30.09	24	30	5.79	5.79	26.26	32.05	28	30
2	59	6.0	SPMS	13	5	5	34	38	5	3	15.03	15.73	28.57	23.89	46	52	15.62	15.69	25.99	18.8	51	50
3	39	6.0	SPMS	8	5	4	68	50	24	17	13.84	11.53	40.44	39.55	30	31	18.41	9.98	47.26	39.28	26	25
4	50	3.5	SPMS	17	3	4	37	47	14	14	5.69	6.08	23.05	26.92	49	45	5.82	6.41	24.89	23.61	50	51
5	53	5.5	SPMS	20	5	5	8	18	5	5	7.67	10.02	24.98	27	52	49	8.06	8.75	24.46	27.3	47	51
6	46	3.5	PPMS	4	3	3	11	17	4	2	5.24	4.97	23.66	25	43	42	5.26	5.22	23.24	22.75	37	44
7	59	4.0	PPMS	16	3	3	30	24	5	4	7.55	6.76	27.72	26.56	46	40	7.31	6.88	29.86	25.91	44	39
8	49	3.5	SPMS	24	3	3	11	19	6	4	5.75	6.5	17.41	19.09	62	70	5.55	6.19	16.87	16.43	68	72
9	59	4.0	SPMS	21	4	4	62	58	29	17	6	5.79	23.35	26.86	38	29	6.04	5.34	23.47	22.33	40	37
10	39	3.5	PPMS	1	3	4	43	34	16	16	4.33	4.6	21.58	22.1	44	42	4.26	4.59	20.57	19.53	44	45
11	61	4.0	PPMS	20	5	5	30	30	20	16	7.71	10.16	35.8	36.82	30	24	8.48	8.4	32.74	32.41	30	24
12	33	3.0	PPMS	2	3	3	36	16	9	8	5.61	5.46	30.04	26.97	49	49	5.63	5.33	27.53	25.78	53	55
13	62	6.0	PPMS	32	4	4	34	27	6	5	6.61	7.95	20.2	25.68	50	43	6.24	6.2	20.6	21.8	48	51
14	62	6.0	PPMS	21	5	5	44	44	7	9	8.45	7.25	73.27	66.94	31	32	7.5	6.96	63.93	64.34	32	25
15	33	1.5	PPMS	4	3	3	10	5	7	12	4.95	4.89	16.82	17.04	54	57	4.9	5.03	16.92	17.66	48	53
16	64	6.0	PPMS	5	5	5	43	44	5	5	16.39	20.25	21.3	21.49	44	45	14.69	15.09	20.88	20.02	45	46

Abbreviations: EDSS, Expanded Disability Status Scale; MS, multiple sclerosis; SPMS, secondary progressive MS; PPMS, primary progressive MS; CGI-S, Clinical Global Impression Severity Scale; WEIMuS, Würzburger Fatigue Inventory for MS; BDI, Deck Depression Inventory; T25W, timed 25-foot walk; SDMT, symbol digit modalities test.

## Data Availability

The data used to support the findings of this study are available from the corresponding author on reasonable request.
